# Parliament and the Profession

**Published:** 1919-03-08

**Authors:** 


### PARLIAMENT AND THE PROFESSION.

### Medical Treatment of Discharged Soldiers.

Sir J. Craig, speaking for the Pensions Minister, stated that all men, whether insured or not, who are invalided from the Forces or certified on demobilisation to be impaired in health by reason of their service, are entitled to free medical benefit under the National Health Insurance Acts—that is, to the services of a general medical practitioner, and the supply of medicines, except in the case of uninsured men whose total income exceeds £160 a year. There is no medical benefit under the Insurance Acts in Ireland, but by special arrangements with the Irish Insurance Commissioners, which came into force in October 1918, provision similar to the above was extended to Ireland so far as invalided men are concerned, and the question of extending the arrangements to men in impaired health on demobilisation is at present under consideration.

### Nursing Sisters in the Royal Navy.

Colonel Wedgwood asked the First Lord of the Admiralty why regular naval nursing sisters were omitted from the recent general increase of pay for all officers and men in the Navy, in view of the official standing order that they are to be regarded as officers of the hospital, taking a position immediately after the surgeons. Doctor Macnamara replied that the question of an increase in pay is being considered, and proposals on the subject will shortly be submitted to the Board. The nursing sisters are engaged and paid on a quasi-civil basis, and their pay is separately dealt with from that of naval officers.

### Nerve-Shaken Soldiers.

In answer to a question put by Major Entwistle, Sir James Craig said that no discharged men suffering from shell-shock or other nervous disorders, unless medically certified as lunatics, are sent to asylum. Shell-shock cases are being treated in homes of recovery of the Ministry of Pensions and Army neurological hospitals. Special accommodation is being provided for shell-shock cases, and it is considered probable that some of the special neurological centres may be taken over by the Ministry.

### Marriage of Assistant Medical Officers in Asylums.

Sir Watson Cheyne asked the Home Secretary whether he was aware that assistant medical officers of public asylums are not allowed to marry, and, if so, whether the rule will be rescinded. Mr. Shortt replied that any rules governing the conditions of service of medical officers of asylums are made by the visiting committees who manage the asylums and appoint the officers. He was informed that there is no general rule preventing marriage, but the possibility is largely dependent on the nature of the residence provided, which in a large majority of cases is only suitable for an unmarried officer. The Board of Control have used their influence in the direction of securing the provision of such accommodation as will enable the senior assistant and, in large asylums, the second assistant to marry. Separate houses have been provided with this object in the grounds of some ten county asylums, including four of those belonging to the London County Council.

